# First person – Alexandra Venuto

**DOI:** 10.1242/bio.059664

**Published:** 2022-10-18

## Abstract

First Person is a series of interviews with the first authors of a selection of papers published in Biology Open, helping researchers promote themselves alongside their papers. Alexandra Venuto is first author on ‘ Alone in a crowd: Effect of a nonfunctional lateral line on expression of the social hormone parathyroid hormone 2’, published in BiO. Alexandra is a PhD Student in the lab of Dr. Timothy Erickson at East Carolina University, investigating the involvement of the mechanosensory lateral line in social behavior and surfacing behaviors in larval zebrafish.


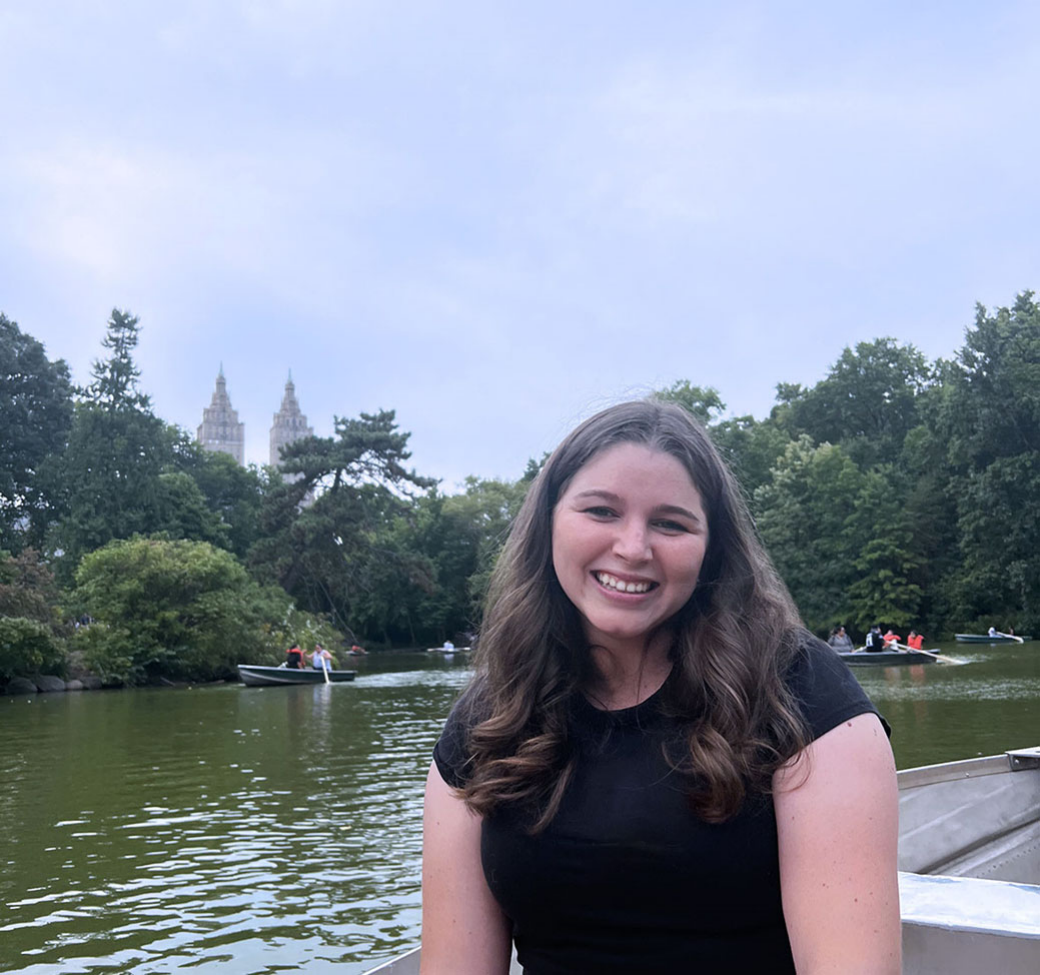


**Alexandra Venuto**

**Describe your scientific journey and your current research focus**

I started my research as an undergraduate student at Villanova University working in genetics and developmental biology where I learned how great it is to use zebrafish as a model organism! I ended up continuing to work with zebrafish in grad school and found additional passions in sensory biology and neuroethology. Currently, I focus on early larval behaviors mediated by the unique lateral line sensory system using a novel genetic mutant.

**Who or what inspired you to become a scientist?**

I really enjoyed my AP Biology class in high school (shoutout Mr. Jennings) and decided to try out biology as my major in college. The first two biology classes I took in college were centered around genetics and were taught by the same professor, Dr. DiBenedetto, who's lab I then joined. Dr. D is a fantastic mentor, who taught me the fundamentals of genetics and instilled thinking of the broader picture while conducting research.

**How would you explain the main finding of your paper?**

The main finding is that larval zebrafish use their lateral line sensory system to detect each other in their environment, which seems to make them feel less alone.

“… larval zebrafish use their lateral line sensory system to detect each other in their environment, which seems to make them feel less alone.”

**What are the potential implications of this finding for your field of research?**

Through this study, we show how lateral line detection of conspecifics has the capacity to specifically modulate neuropeptides in the brain. We also offer a new transgenic line to track parathyroid hormone 2 dynamics in live zebrafish, which can lead to greater understanding of the neuropeptide.

“… we show how lateral line detection of conspecifics has the capacity to specifically modulate neuropeptides in the brain.”

**Figure:**
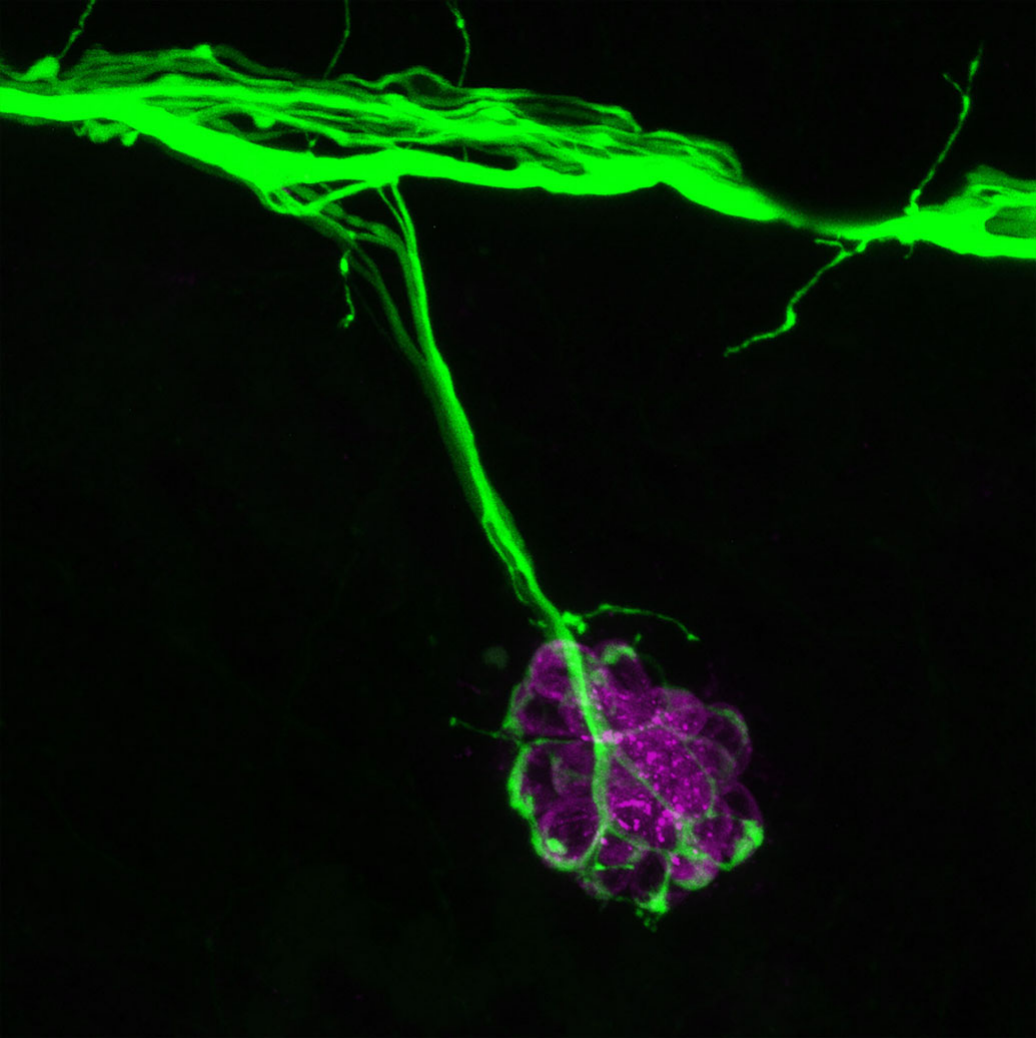
**The mechanically sensitive hair cells of the lateral line are distributed along the body in clusters called neuromasts.** This image shows a single neuromast innervated by neuronal differentiation 1 (neuroD). This image was taken using live transgenic zebrafish expressing green fluorescent protein (shown in green), and hair cells were labelled with FM 4-64 dye (shown in magenta).

**Which part of this research project was the most rewarding?**

My PI (Dr. Erickson) moved to the University of New Brunswick in Fredericton, Canada, in the middle of this project and after some deliberation, I decided to stay behind at ECU. So, honestly, the most rewarding part of this project was realizing how closely we could continue to work together successfully despite being in different countries. This paper blends people from his new lab with people from his previous lab and it has been awesome to come together through this project!

**What do you enjoy most about being an early-career researcher?**

I enjoy getting to know and meet some of the spectacular researchers that I often cite and base my own experiments from. Especially since COVID hit in the middle of my PhD, it is great to attend conferences and make connections again that I may have taken for granted in the beginning of my grad school experience.

**What piece of advice would you give to the next generation of researchers?**

I would say to keep your mind open to a variety of research paths. I think it is easy to bury yourself in your specific niche, but I've often found that the best ideas come when you are reading or talking about research that is unique from your own.

**What's next for you?**

I plan to start my journey as a postdoc next year in sensory biology. Ultimately, I would like to be a professor at a smaller institution where I can teach and train young researchers in the field of neuroethology.
